# Investigation of the support constraint effect and failure instability law of tunnels constructed using the New Austrian tunneling method

**DOI:** 10.1038/s41598-022-09826-1

**Published:** 2022-04-06

**Authors:** Dongxu Chen, Laigui Wang, Chuang Sun, Chengzhi Jia, Luxin Zheng

**Affiliations:** 1 School of Mechanics and Engineering, Liaoning Technical University, Fuxin, 123000 Liaoning People’s Republic of China; 2 School of Civil Engineering, Liaoning Technical University, Fuxin, 123000 Liaoning People’s Republic of China; 3 Dalian Branch, China Railway No. 9 Group Co. Ltd, Dalian, 116000 Liaoning People’s Republic of China

**Keywords:** Geophysics, Solid Earth sciences, Petrology

## Abstract

The application of a reasonable numerical calculation method is the key to accurately analyzing tunnel rock-support interactions. In this paper, we address the support constraint effect of tunnels and analyze the influence of related factors based on the confinement convergence method. Rupturable support models are developed using FLAC^3D^ to intuitively show the numerical calculation results of tunnels. The results imply that the virtual supporting force generated by the support constraint effect should be considered in two-dimensional rock tunnel model calculations, and that the supporting force of the support should be increased by 2–3% of the maximum supporting force. Boundary effects should be considered in the three-dimensional tunnel model calculations, in which the influence range of the model boundary effect is nearly 1.5 times the tunnel span. A comparison of the field monitoring data and numerical calculations of the Baoshan tunnel project shows that the numerical results that consider the support constraint effect are in better agreement with the actual project situation. The rupturable support models can also reflect the stress and failure evolution law of supports, and provide support for the accurate evaluation of tunnel engineering stability.

## Introduction

Numerical analysis and the confinement convergence method (CCM) are the main tools used to analyze the stability of the New Austrian tunneling method (NATM) project. However, tunnel rock-support interactions under complex engineering conditions remain a complicated issue within engineering stability analysis^1^. At present, the initial support design of the NATM mainly relies on engineering analogies and engineering experience, and has been verified by a comparison of field monitoring data and numerical calculations. Calculations within mainstream numerical analysis are mainly performed using two-dimensional (2D) numerical models to analyze the stability of the surrounding rock and tunnel support. However, tunnel rock-support interactions cannot be truly represented owing to limitations of the current support model and CCM. An in-depth study on the interaction principle between the surrounding rock and support in three-dimensional (3D) tunnel space is therefore required to propose a reasonable numerical calculation method for tunnel surrounding rock support, which is of great scientific significance for accurately assessing tunnel surrounding rock stability under complex conditions.

In recent years, many scholars have carried out a lot of research on CCM. For example, through theoretical analysis and a large amount of engineering experience, Oreste^2, 3^ derived mechanical characteristic equations to describe a variety of tunnel supports. Su et al.^4^ proposed an analytical method to calculate the factor of safety of the surrounding rock component from the perspective of considering the surrounding rock as one of the support components. Kabwe et al.^5^ applied the CCM to study the responses of elastic-perfectly plastic ground in circular tunnels under the control of Hoek–Brown and Mohr–Coulomb criteria. Based on numerical analysis, Oke et al.^6^ proposed a methodology and solution to improve the applicability of the CCM for deep buried tunnels. Paraskevopoulou et al.^7^ introduced a time factor into the CCM to improve its engineering applicability. Carranza-Torres et al.^8^ derived equations for closed round steel-supports in the CCM and analyzed block angle effects on the support bearing capacity and stiffness. Through numerical simulation and theoretical analysis, Cui et al.^9^ conducted two-stage analyses on support-rock interactions using a numerical procedure. The procedure accounts for the strain-softening behaviour of the rock mass and the effect of delayed support installation.

In terms of numerical simulation of tunnel stability, Do et al.^10^ established a 3D numerical model to evaluate the effects of mutual disturbance on the support stability during double-hole tunnel construction. Du et al.^11^ applied numerical methods to study the effects of different parameters, including geometry and horizontal and vertical loads of U-shaped supports, on the supporting force. Gao et al.^12^ presented new correlations for estimating the surrounding rock pressure of symmetrically shaped tunnels by using numerical calculation method. Li et al.^13^ proposed a new method to evaluate the face stability of rock tunnels beneath the water table using a combination of numerical simulations and a kinematic approach to limit analysis. Berkane et al.^14^ analyzed the failure law of the T1 tunnel of the Algerian East–West Highway under the influence of seismic waves by numerical calculation method. Mayoral et al.^15^ used numerical methods to study the stability of the tunnel during Puebla-Mexico earthquakes. Taking the diversion tunnel in Sri Lanka as the engineering background, Golian et al.^16^ studied the influence of underground water on tunnel stability through numerical calculation method. Wang et al.^17^ developed the concept of critical instability of segmented lining structures to accurately determine the critical instability point. Li et al.^18^ performed a secondary development of FLAC^3D^ to further improve the support arch and anchor modules. Guo ^19^ carried out reliability analysis on the support lining of a circular tunnel excavated under spatially changing conditions.

To sum up, scholars from all over the world have carried out extensive research on CCM, but few of them consider the constraint effect of support on surrounding rock. And the combined support model used in numerical calculation can not accurately judge whether the supporting system is unstable. In this paper, the support constraint effect on surrounding rock is studied based on the CCM, and the calculation method of support constraint effect is given. A rupturable liner (RL) model and rupturable cable (RC) model are developed using FLAC^3D^ and verified by laboratory tests. The Baoshan Tunnel is taken as the engineering background. The influence of support constraint effect on engineering calculation and the application effect of rupturable model in tunnel stability analysis are studied. And the results are compared with field monitoring data to further optimize the tunnel stability evaluation method. Our findings provide an important reference for the support design and stability analysis of complex tunnel engineering.

### Confinement convergence method and support constraint effect

During underground tunnel construction, the working face has a constraint effect on the surrounding rock after excavation, which gradually weakens as the working face advances and the surrounding rock pressure is slowly released^20, 21^. Similar to the constraint effect of the working face, the support structure applied in a tunnel also has a certain constraint effect on the surrounding rock in front of it, which restricts surrounding rock deformation and is herein referred to as the “support constraint effect”. Although the support constraint effect generally exists in tunnel engineering calculations and analysis, it is often ignored. In this paper, the support constraint effect is studied using numerical calculation methods, and the tunnel engineering stability analysis method is further optimized.

#### Calculation model of support constraint effect

**Figure 1:**
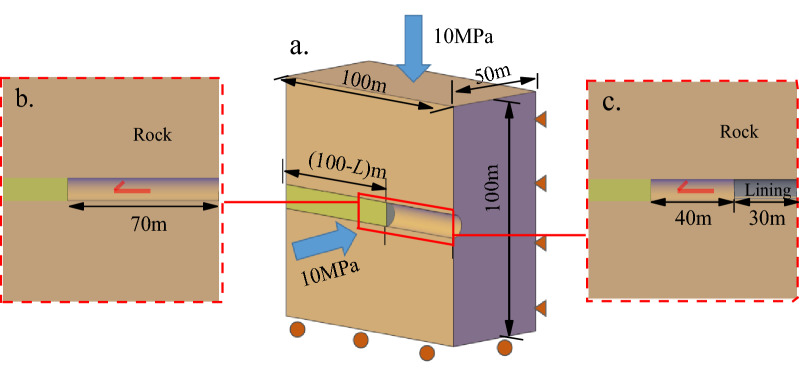
Characteristics of the support constraint effect.

In order to fully show the influence of support constraint effect on the analysis of tunnels, a hypothetical circular tunnel is analyzed in this section. The tunnel radius *R* = 5 m and initial rock stress *σ*_0_ = 10 MPa. We use the Mohr-Coulomb elastoplastic model for calculation, and the surrounding rock parameters are listed in Table 1. The tunnel is excavated using the full section method with a round length of 2 m and finally excavated to 70 m. The tunnel model is shown in Fig. 1a.

**Table 1:** Rock mechanical parameters of tunnel surrounding rock.

Parameter	*E*/GPa	*μ*	*σ*_*c*_/MPa	*c*/MPa	*Φ*/^o^
peak	15.0	0.26	35.0	4.0	38.0
residual			6.0	2.0	30.0

*E*: Young’s modulus; *μ*: Poisson’s ratio; *σ*_*c*_: Compressive strength of intact rock; *c*: Cohesive force; *Φ*: Internal friction angle.

We assume two construction conditions. The first is that no support is applied during tunnel excavation, as shown in Fig. 1b. The second is that 30m lining is applied during tunnel excavation, as shown in Fig. 1c. The elastic modulus of the lining *E*_c_ = 21.5 GPa, the Poisson’s ratio *μ*_c_ = 0.25, and the thickness is set to 15 cm, 20 cm and 25 cm. The FLAC^3D^ numerical calculation software is used to calculate the conditions. After the calculation, longitudinal deformation profile (LDP) curves of the surrounding rock are then drawn.

#### Analysis of support constraint effect

**Figure 2:**
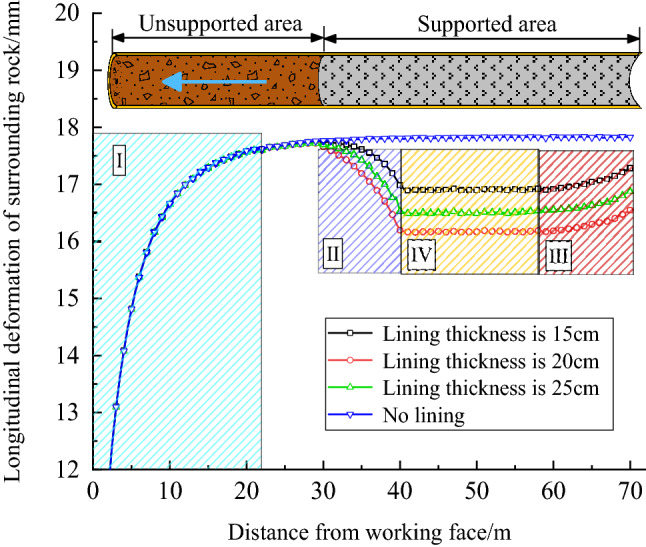
Support constraint effect for different supporting strength conditions.

The calculation results are shown in Fig. 2. When no lining is applied to surrounding rock, the deformation of surrounding rock near the working face increases gradually due to the constraint effect of working face (shaded region I), and the deformation of surrounding rock reaches stability beyond the influence range of constraint effect of working face. When a section of lining is applied to the surrounding rock, the surrounding rock near to the working face is also constrained by the working face. When the surrounding rock exceeds the constraint range of the working face, the surrounding rock stays stable first. However, the surrounding rock deformation gradually decreases in the area adjacent to the lining (shaded region II), and finally reaches stability in the support area (shaded region IV). This is because the lining not only directly limits the deformation of the supported surrounding rock, but also provides virtual supporting force for the unsupported surrounding rock (shaded region II), indicating that the support has a constraint effect on the surrounding rock in front. In the boundary region (shaded region III), due to the boundary effect, the deformation rebounds and the original stable deformation gradually becomes larger. This is because there is no lining outside the boundary of the model, so the closer it is to the boundary, the less obvious the support constraint effect is. Finally, the support constraint effect is completely lost at the boundary of the model.

By comparing the supporting curves, it can also be found that although the lining thickness is different, the influence range of support constraint effect is similar. When the lining thickness is 15 cm, 20 cm and 25 cm, the influence range of the support constraint effect is about 10 m, and the influence range of the boundary effect is about 15 m (1.5 times the tunnel span). In order to make the calculation faster, we often use a 2D model for tunnel stability analysis. Because the support constraint effect is ignored, the deformation calculated by the 2D model is the same as the 3D model boundary, while the actual deformation of surrounding rock should be the same as that in the shaded region IV. Therefore, the virtual supporting force provided by the support constraint effect should be added to the 2D model. The calculation method of virtual supporting force is shown as follows^2^.

1 $$P{ = }K_{{\text{s}}} \cdot \frac{u}{{r_{{\text{o}}} }}$$

where *P* is the supporting force of the support, *K*_s_ is the stiffness of the support, *u* is the radial deformation of the support, *r*_o_ is the tunnel radius. The following equation can be transformed from formula (1).

2 $$S{ = }\frac{\Delta P}{{P_{{\text{b}}} }} \times 100{\text{\% }} = \frac{{u_{{\text{b}}} - u_{{\text{c}}} }}{{u_{{\text{b}}} }} \times 100{\text{\% }}$$

where *S* is the ratio of the virtual supporting force to the supporting force at the model boundary, D*P* is virtual supporting force, *P*_b_ is the supporting force at the model boundary, *u*_b_ is the deformation amount of surrounding rock at the model boundary, *u*_c_ is the deformation amount of surrounding rock in shaded region IV.

Put the data in Fig. 2 into equation (2) for calculation. The calculation results show that although the supporting force of the support is different, the ratio *S* is similar, which is about 2–3% of the supporting force at the model boundary. When the lining thickness is 15cm, the *S* is about 2.6%. When the lining thickness is 20cm, the *S* is about 2.3%. When the lining thickness is 25cm, the *S* is about 2.8%. Therefore, in order to make the calculation result more close to the actual situation, it is suggested to add virtual supporting force in the calculation of the 2D model, that is, to increase the supporting force by 2–3%.

### Secondary development of the lining failure model

Built-in liner units are often used to simulate concrete lining in FLAC^3D^, but the liner unit is treated as an elastomer in the calculation process, which cannot simulate cracking and spalling failure of the lining in practical engineering. This paper improves the liner unit using FISH programming language based on FLAC^3D^ according to the stress and failure characteristics of the field lining. The RL model is established by setting the yield failure criterion of the liner unit, which makes the numerical calculation results of the tunnel lining structure more intuitive.

#### Program implementation of the RL model

To better reflect the stress state and failure law of the lining in the numerical calculation and improve the accuracy of the lining stability analysis, this paper modifies the liner unit based on FISH programming language and constructs the RL model. The specific process is as follows^22^.

1. The tunnel model is constructed using FLAC^3D^, the lining units are constructed at the tunnel section, and the ID (identity) of the lining unit is defined as “1”, as shown in Fig. 3a. As mentioned, the lining units together form a support body at this time and are connected through nodes to prevent fracture failure. The coordinates of all of the unit nodes with “1” values are extracted using FISH, the data are stored in a function table, and the lining unit “1” is then deleted.

**Figure 3:**
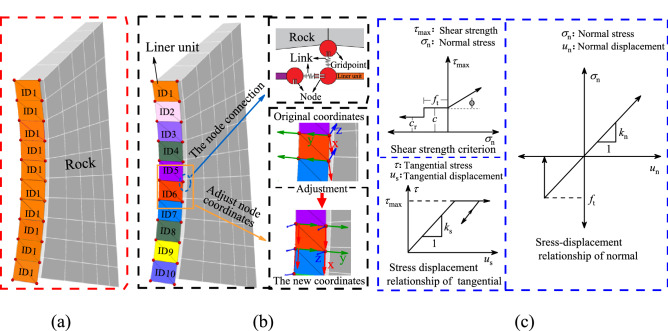
Construction process and mechanical model of the RL units.
2. The node coordinates of all lining units are extracted in the function table, the lining units are constructed with different ID values using the four-point coordinate method, and the ID is defined as n, (n = 1, 2…), as shown in Fig. 3b. At this time, there is an independent lining unit on each surrounding rock unit of the tunnel excavation section. Owing to the different IDs, the two lining units are not connected with each other and two nodes of lining “n” and “n + 1” units overlap at the lap position, and so on.
3. All of the links between the lining unit and surrounding rock nodes are deleted. The nodes of the “n + 1” unit with a rock unit are connected using FISH and the nodes of the “n” unit with “n + 1” unit nodes are connected to establish new links at the node lap positions. The two lining units at this lap position are thus linked with the rock unit and with each other, thus the force transfer between the lining units and between the lining unit and rock unit is realized, as shown in Fig. 3c.
4. The coordinate system must be adjusted because the local coordinates of each node change with the tunnel model coordinate system when establishing the lining unit. The local coordinate system of each node is unified by FISH, in which the connection direction of the lining unit nodes is defined as X, the direction along the tunnel excavation direction is defined as Y, and the radial direction of the tunnel section is defined as Z. The transformation process is shown in Fig. 3c.
5. A connection value is assigned to the link between nodes, and the stress change of the link is monitored in the calculation. When the pressure or tension of the link exceeds the limit value of the concrete lining, the link is deleted by FISH such that the connection between the units is broken to simulate the lining fracture process.

#### Comparative analysis of RL model test

To test the rationality of the RL model, compressive strength, flexural strength, and splitting tensile strength experiments were carried out in the laboratory using concrete samples. The RL model was used to simulate the experiments according to the obtained parameters, and then compared with the experimental results. The concrete ratio was set to cement: water: sand: stone: accelerator = 1:0.51:1.82:1.57:0.06 according to existing engineering data.

Three groups of cubic specimens with dimensions of 150 × 150 × 150 mm and cuboid specimens with dimensions of 150 × 150 × 550 mm were poured and allowed to cure for 14 days in a curing box. A TAW-2000 computer controlled electro-hydraulic servo rigid pressure testing machine was used to perform compressive and flexural experiments on the concrete samples. The results indicate a concrete elastic modulus *E*_c_ of 21.5 GPa, Poisson’s ratio μ_c_ of 0.25, compressive strength of 25.8 MPa, tensile strength of 2.96 MPa, and flexural strength of 4.32 MPa.

**Figure 4:**
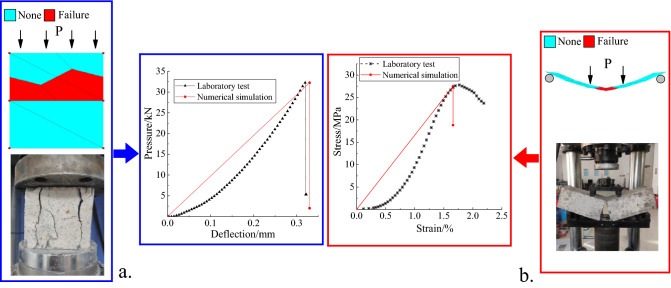
Comparison of the concrete test results.

The RL model was constructed using FLAC^3D^ to simulate the compression and flexural experiments of concrete according to its rock mechanical parameters. A comparison between the simulations and experiments is shown in Fig. 4.

The simulation curve was obtained by gradually increasing the load P and comparing with the experimental curve. Figure 4a shows that the failure load was 4.30 MPa during the concrete bending test simulation, which is similar to 4.32 MPa obtained in the experiments, yielding an error of 0.46%. As shown in Fig. 4b, the failure load was 27.5 MPa during the concrete compression test simulations, which is similar to 27.8 MPa obtained from the experiments, yielding an error of 1.08%. The comparison of experimental and numerical results therefore verifies that the RL model can simulate the lining structure failure characteristics.

### Secondary development of the anchor failure model

The cable model simulating anchors was built in FLAC^3D^ and the connection between the nodes of the cable units were treated as rigid and unbreakable. However, anchor breakage failure often occurs in practical engineering, especially in deep caverns. There is thus is a large deviation between the cable units simulation results and real situation. In this paper, the secondary development of the cable units were carried out based on FLAC^3D^ embedded in FISH language. The RC model was established upon setting the anchor breakage criterion, which improves the calculated stress and failure characteristics of the anchor to be in better agreement with those in engineering practice.

#### Program implementation of RC model

To study the stress state and failure evolution law of anchors and improve the accuracy of anchor stability analysis, this paper modifies the cable unit based on the FISH programming language of FLAC^3D^ and constructs the RC model. Cable model as shown in Fig. 5a. RC model as shown in Fig. 5c. The specific process is as follows

1. A tunnel model is developed using FLAC^3D^. The anchor starting position is selected on the surrounding rock according to the engineering design, which includes the actual drilling position, and the anchor direction is determined. The length of a cable unit is constructed at starting point #1, and the ID of the cable unit is defined as “1”, as shown in Fig. 5b. A cable unit of the same length is similarly constructed along the anchor drilling direction at starting point #2 and the unit’s ID is defined as “2”. The cable units for the other anchor positions are constructed in the same manner, and the unit IDs are sorted in sequence. All anchor units are referred to as the “first batch” at this time.

**Figure 5:**
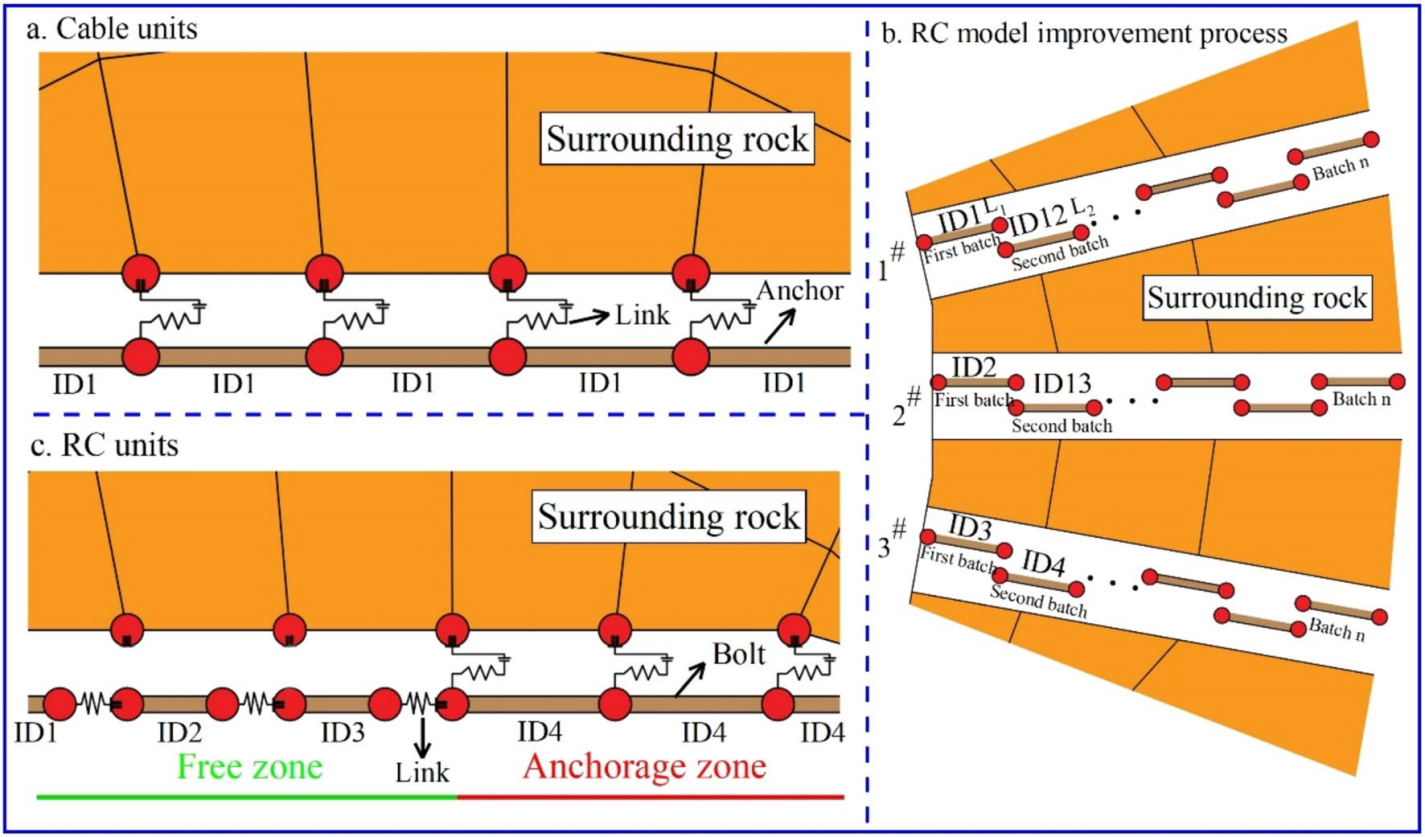
Construction process and mechanical model of the RC units.
2. A length of cable unit is constructed from points L_1_ to L_2_ along the anchor direction, and the unit ID is immediately sorted following the unit ID in step (1). Similarly, the remaining cable units are immediately constructed following the “first batch” cable units and with the same length along the anchor drilling direction. The unit IDs continue to be sorted. The cable units constructed in this stage are called the “second batch”. The number of batches n is controlled according to the engineering accuracy requirements. More batches built within a certain distance are associated with higher calculation accuracy, and the calculation cost accordingly increases.
3. All of the cable units at this point are connected to the zone through the link. All of the links are then deleted except those of the “n batch”, and the group of reserved links is defined as the “grout-link”.
4. A link is constructed between the cable units, as shown in Fig. 5c. Links are constructed between the cable unit nodes using the “structure link create target node range structure-type cable” command. At this point, both nodes build links to each other, in which two nodes are connected by two links. However, this does not conform with the FLAC^3D^ calculation criteria and the software will report an error during the model calculation. In this case, two link IDs are required: one odd and one even. All of the links are deleted with odd or even IDs according to the parity law, thus there is only one link between the two nodes. The link built at this stage is defined as the “cable-link”.
5. The links grouped as “grout-links” are connected with the surrounding rock. The anchor and grout parameters are assigned to this group to simulate the anchor section. The links grouped as “cable-links” are used to connect the cable unit nodes. The group of links is rigid at this time. The links are set as the normal yield along the x-direction and the yield parameters are given. The anchor parameters are given to the cable units to simulate the free section.
6. The link stress can be monitored during the calculation by assigning a value to the links between the nodes. When the tensile force of the cable exceeds the limit value of the anchor, the link is deleted using FISH after running a certain number of steps to simulate the yield fracture process of the anchor.

#### Comparative analysis of RC model test

To verify the rationality of the RC model, full-length bonded anchor pull-out experiments were carried out in the laboratory, the RC model was used for simulations based on FLAC^3D^, and the results are compared. In the pull-out experiment, 14-mm-diameter reinforcement is used as the anchor, granite is used as the surrounding rock, and cement mortar is used as the grout. The rock mechanical parameters of the experimental materials are listed in Table 2.

**Table 2:** Rock mechanical parameters of pull-out test materials.

Material	Anchor	Mortar	Grout
Properties	HRB400	1:2	–
Modulus of elasticity/GPa	200	5.77	13.27
Poisson's ratio	0.31	0.33	0.22
Cohesion/MPa	–	8.4	3.5
Internal friction angle/°	–	39	36
Yield strength/MPa	456	–	–
Compressive strength/MPa	–	28.7	58.2
Tensile strength/MPa	–	2.4	6.2

**Figure 6:**
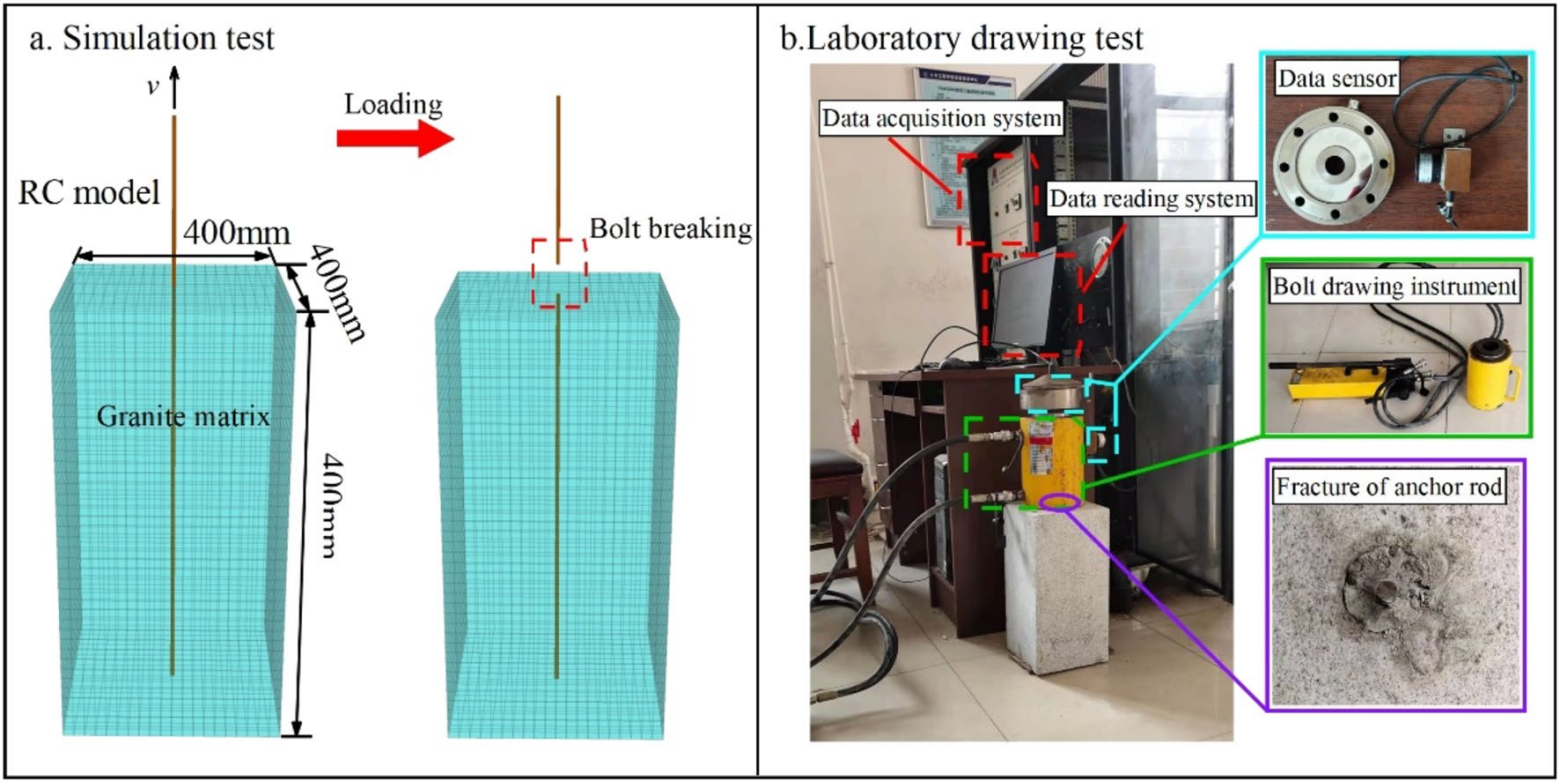
Comparison of pull-out test loading process.

The RC model is used to build the experimental model and carry out anchor drawing simulations according to the rock mechanical parameters in Table 2. A comparison between the simulations and experiments is shown in Fig. 6.

**Figure 7:**
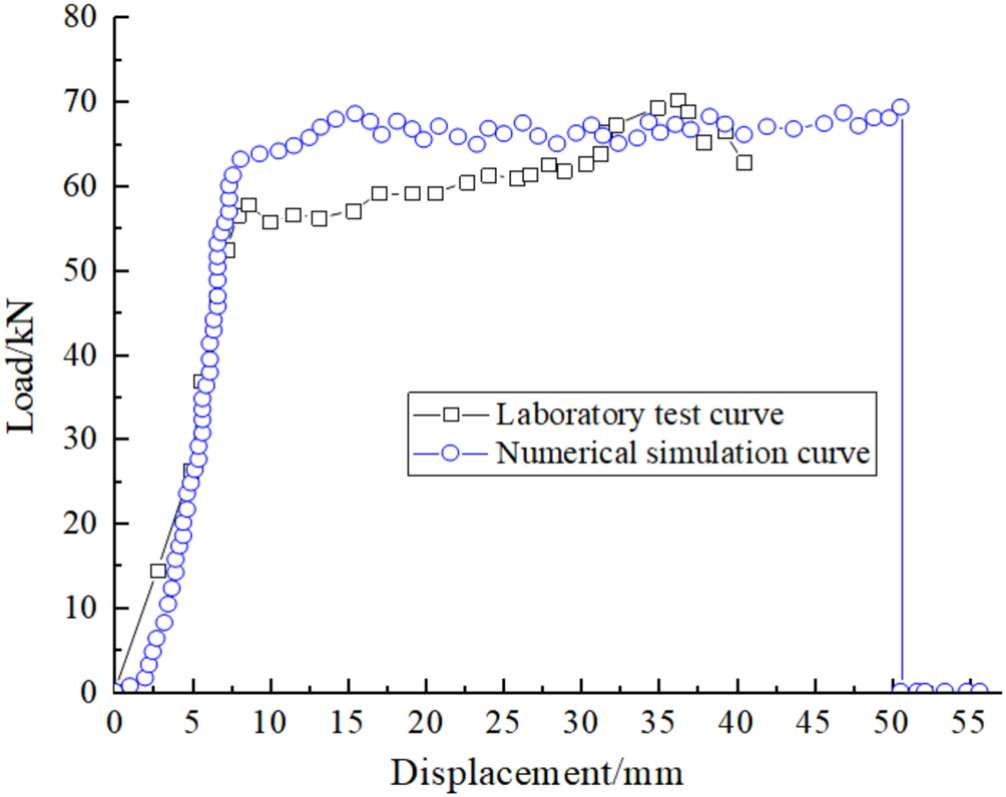
Comparison curve of the pull-out test results.

A comparison of the experimental curves is shown in Fig. 7. The numerical simulation results show that the load-displacement curve follows a linear relationship during the initial stage of gradually increasing deformation. When the load reaches 63.1 kN and the displacement reaches 8.2 mm, the load changes to a gentle upward trend. Once the load reaches 69.8 kN and the displacement reaches 50.6 mm, the load decreases to 0 kN and the anchor breaks. The trend of the simulated curve is essentially the same as the experimental curve. The maximum failure load simulated by the RC model is 69.8 kN, which is similar to the real anchor failure load of 70.6 kN, with an error of 1.1%. This implies that the RC model can demonstrate the deformation and failure characteristics of a real anchor, which is more suitable for practical engineering than the original cable model.

### NATM engineering case analysis

In order to explore the significance of the results of the support constrain effect and rupturable support models, this paper takes the Baoshan Tunnel as the engineering background and discusses the application of the above work.

#### Project overview

**Figure 8:**
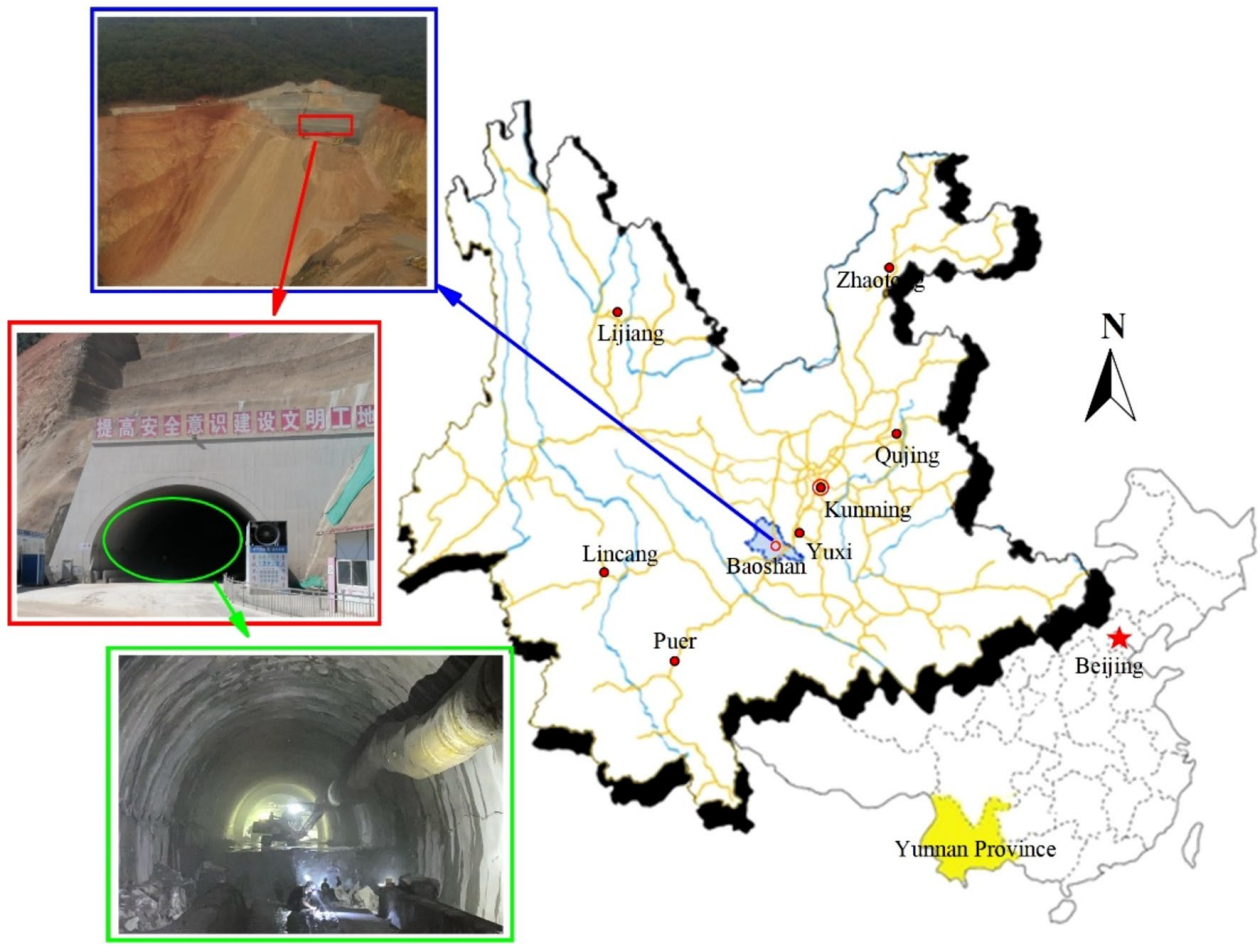
Overview of the Baoshan Tunnel.

The Baoshan Tunnel is located in Baoshan Village, Eshan County, Yuxi City, Yunnan Province, China, with a length of 1640 m and maximum burial depth of 132 m. The tunnel area belongs to tectonic denudation middle mountain landform, has large topographic fluctuations, and most of the surrounding rocks are grade IV. The tunnel body section is mostly interbedded medium argillaceous sandstone and sandstone with slightly developed rock joints, fissures, and groundwater. The tunnel outlet section is mainly Quaternary gray shale, breccia limestone, gravel, and silty clay with uniform thickness. The tunnel was excavated via the step method with a step length of 6 m. The upper step is 0.6 m from the working face, and anchors (mortar anchors, length = 2.5 m, spacing = 1.2 m, row spacing = 1.2 m) and I21 steel arch frames (shed spacing = 0.6 m) were applied. The lower step was sprayed with concrete to form a closed ring with a concrete thickness of 0.2 m. The tunnel geology and construction overview are shown in Fig. 8.

Sandstone samples were obtained by onsite drilling and the rock mechanical parameters of the intact sandstone were obtained using a TAW-2000 computer controlled electro-hydraulic servo rigid pressure testing machine. According to the laboratory test data and field engineering investigation results, the GSI (geological strength index)^23–25^ of the surrounding rock ranges between 42 and 48. The Hoek-Brown strength parameters are converted into equivalent Mohr-Coulomb peak and residual strength parameters^26^. The rock mechanical parameters of the surrounding rock are listed in Table 3.

**Table 3:** Rock mechanical parameters of pull-out test materials.

*γ*/kN/m^3^	*E*/GPa	*μ*	Peak intensity		Residual strength	
			*c*^*p*^/MPa	*Φ*^*p*^/o	*c*^*r*^/MPa	*Φ*^*r*^/o
24.6	18.48	0.27	3.1	34	0.8	25

*γ*: Weight density; *c*^*p*^: Peak cohesive; *Φ*^*p*^: Peak internal friction angle; *c*^*r*^: Residual cohesion; *Φ*^*r*^: Residual internal friction angle.

#### Numerical calculation model

**Figure 9:**
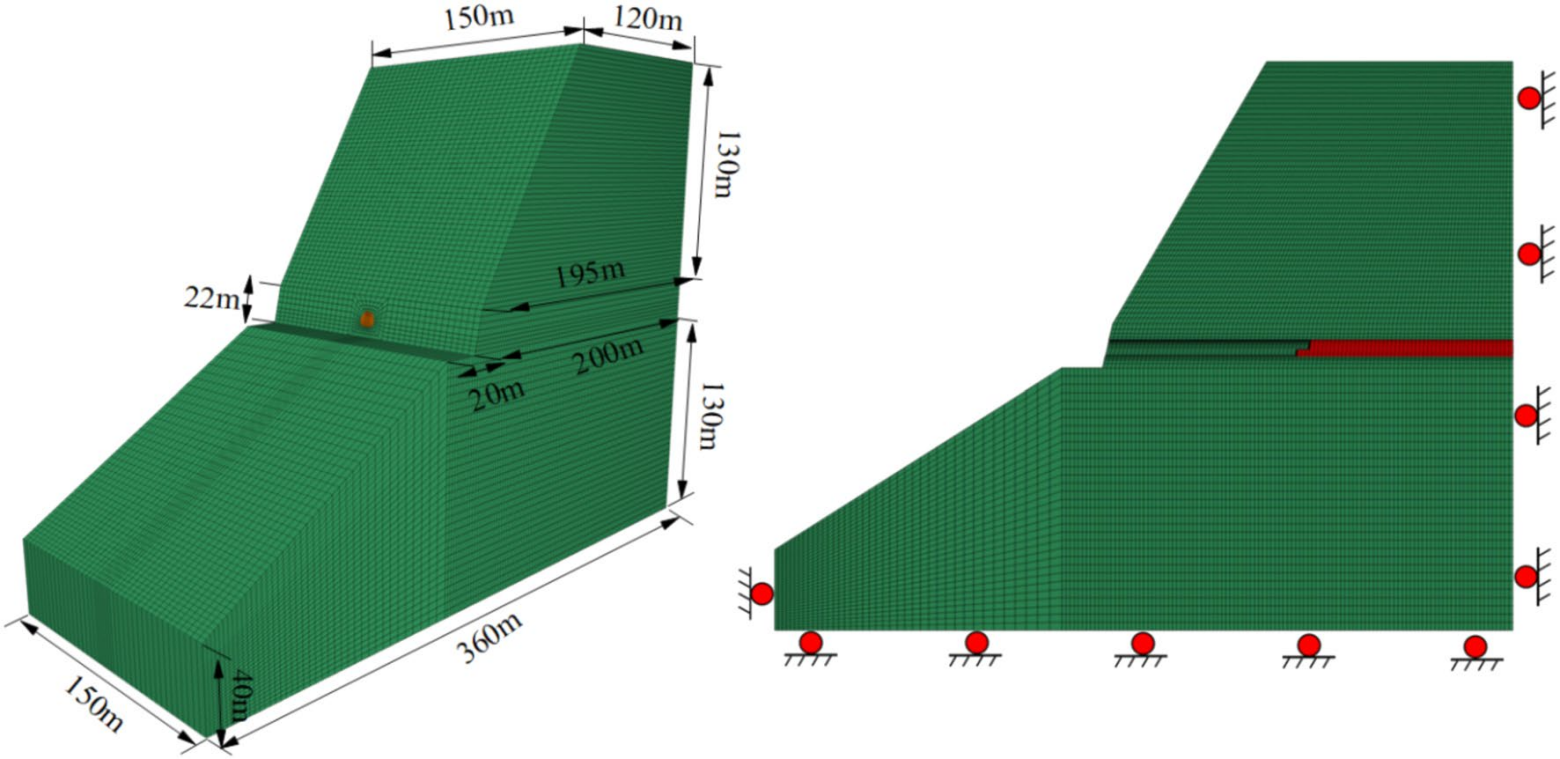
Model size and boundary condition.

The boundary conditions and dimensions of the tunnel numerical model are shown in Fig. 9. Because the maximum burial depth of the tunnel is 132 m, it is not necessary to set an equivalent load at the surrounding rock boundary. The surrounding rock is loaded by the weight of the overlying rock. The horizontal direction and bottom of the model are constrained by the displacement boundary, which is generally consistent with the actual boundary conditions. To study the support constraint effect, the model is set to 200 m along the tunnel axial length, the total excavation length is 120 m, and the excavation length is 0.6 m per cycle. The Mohr-Coulomb elastoplastic constitutive model was used for calculation, and the anchor was simulated using the RC model, the lining was simulated by the RL model, and the steel arch was simulated by the beam model.

#### Stress characteristics and failure evolution law of the support structure

**Figure 10:**
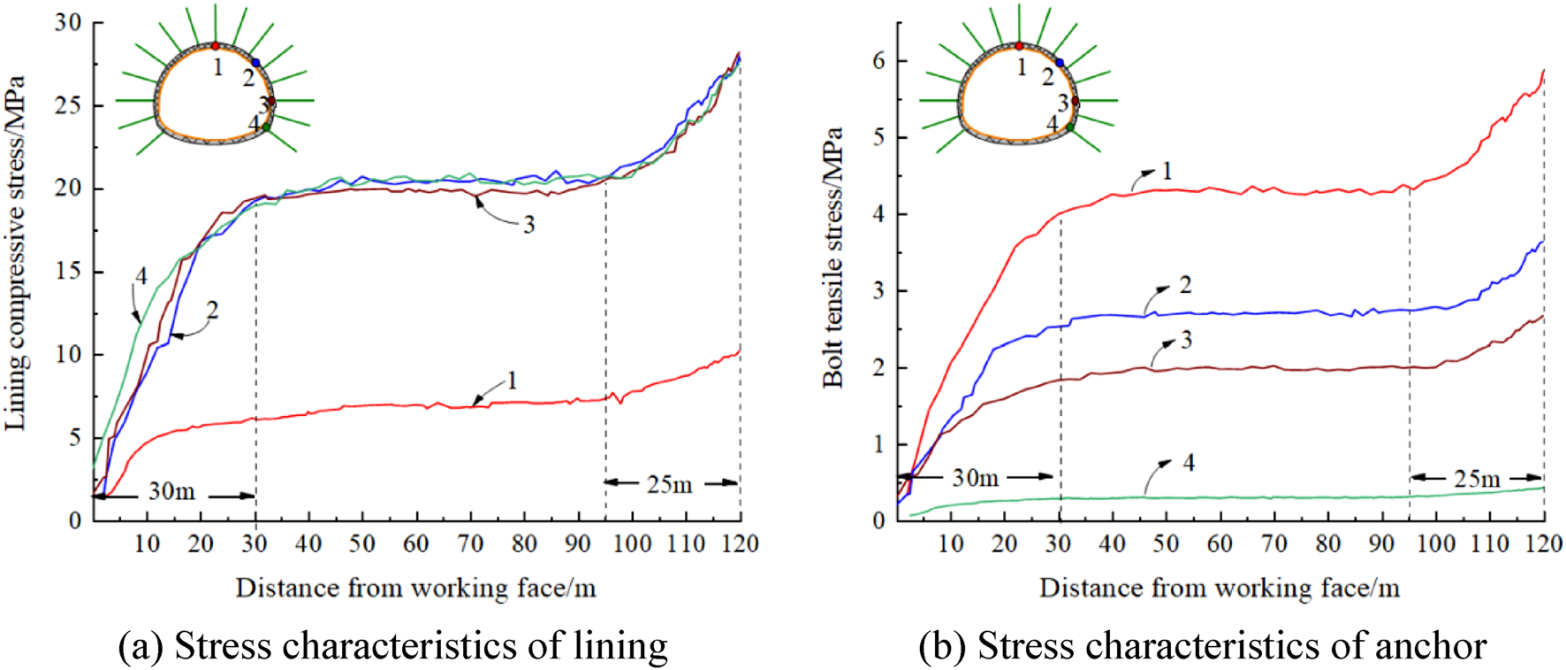
Stress characteristics of the lining and anchor of the Baoshan Tunnel.

Four monitoring positions were selected in the tunnel section, and the stress distribution law of the lining and anchor at the monitoring points was obtained from the calculations. The results are shown in Fig. 10, in which the influence range of the working face constraint effect is approximately 30 m and that of tunnel boundary effect is approximately 25 m, which is approximately 1.5 times the tunnel span. At the boundary position, the compressive stress of the lining at points 1–4 is 10.2, 27.9, 28.2, and 27.5 MPa, respectively. The tensile stress of the anchor at points 1–4 is 5.8, 3.6, 2.7, and 0.4 MPa, respectively. When the boundary effect is exceeded, the compressive stress of the lining at points 1–4 is 6.8, 20.2, 19.8, and 20.5 MPa, respectively, and the tensile stress of the anchor at points 1–4 is 4.3, 2.7, 2.0, and 0.3 MPa, respectively.

**Figure 11:**
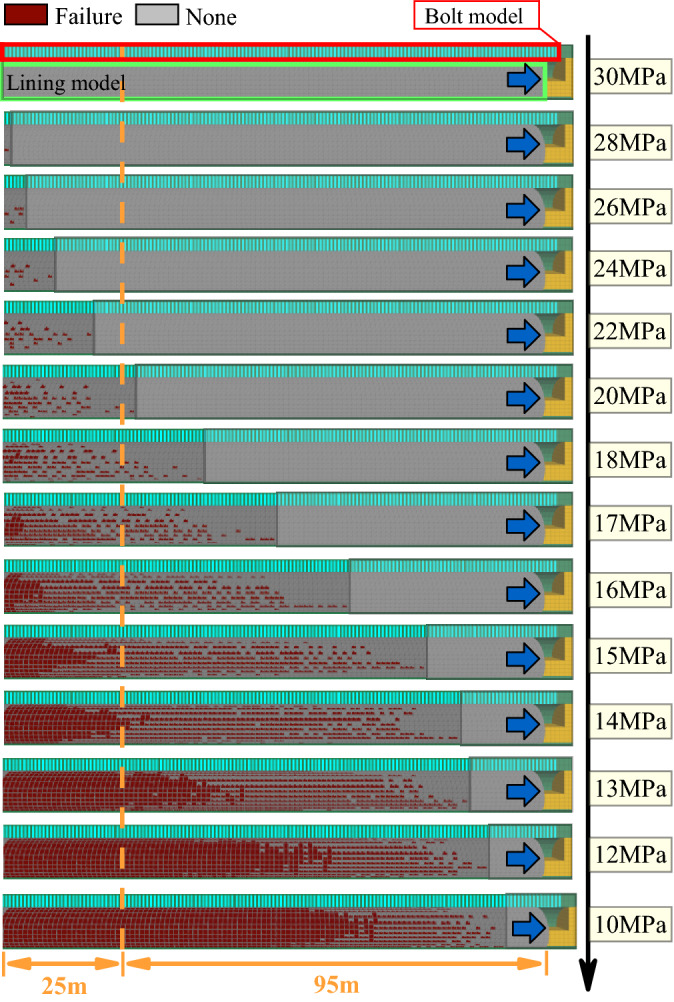
Failure evolution law of the Baoshan Tunnel support.

The tunnel burial depth is shallow and the ultimate anchor deformation is large, thus the anchor generally does not fracture. The lining failure law can be obtained by setting different lining strengths, as shown in Fig. 11. When the concrete lining strength is 30 MPa, the lining structure is essentially undamaged. As the lining strength decreases, the failure area gradually appears within the range of 1–25 m from the model boundary. When the lining strength is less than 20 MPa, the damage range of the lining exceeds 25 m, is affected by the boundary effect, and gradually expands in the tunnel excavation direction. When the lining strength is 18 MPa, local failure occurs at the arch foot of the lining, but the failure range is small. The damage range of the lining gradually increases with decreasing lining strength. When the lining strength is 17 MPa, there are different degrees of damage at the arch waist and spandrel. When the concrete strength is below 14 MPa, the lining structure is damaged over a large area, which indicates that it is difficult for the lining strength to meet the support requirements of the tunnel surrounding rock.

### Field monitoring analysis

**Figure 12:**
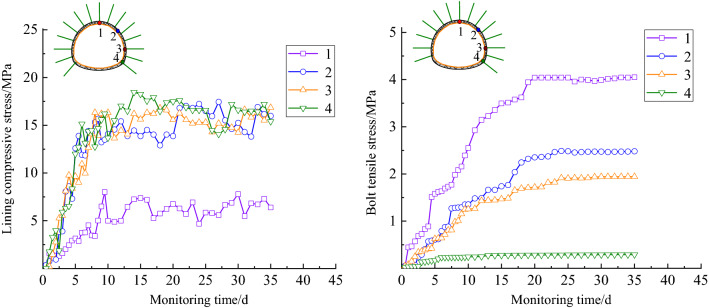
Field monitoring curve of the Baoshan Tunnel.

The concrete lining and anchor of the ZK2+110 section were monitored using HGLJ vibrating wire concrete stress meters and VWRF vibrating wire anchor stress meters. Figure 12 shows the field monitoring positions and monitoring curve. The field monitoring data show that the compressive stress is high in the lining of the tunnel arch shoulder, arch foot, and arch waist, with a maximum value reaching close to 19.4 MPa, and the stress of the tunnel vault lining is small. The anchor tensile force is related to the deformation of surrounding rock. The tensile force on the tunnel vault anchor is the largest, with a maximum value of approximately 4.4 MPa. The tensile stress on the anchor at the arch shoulder and arch waist is small, whereas that at the arch foot is the smallest, with a maximum value of 0.3 MPa. The field monitoring data show that the lining structure is relatively stable without damage. The anchor support system can thus effectively control the loose rock mass without anchor slippage.

If the boundary effect is not considered in the numerical calculation, the maximum internal forces of the lining and anchor at the boundary are 28.2 and 5.8 MPa, respectively. The actual monitoring data show that the maximum compressive stress of the lining is 19.4 MPa and the maximum anchor tension is 4.4 MPa. The numerical results are clearly inconsistent with the actual data. When considering the boundary effect, the maximum internal forces of the lining and anchor obtained by the numerical calculation are 20.5 and 4.3 MPa, respectively, which are in better agreement with the field monitoring data. The numerical calculation results are therefore more consistent with the actual engineering situation when considering the support constraint effect.

## Conclusion

This paper addresses the support constraint effect and analyzes the related influencing factors based on the NATM. The RL and RC models are developed using FLAC^3D^, and the rupturable model is verified by laboratory tests. The stress distribution and failure evolution law of tunnel lining and anchor support structures are studied taking the Baoshan tunnel as the engineering background. The main conclusions are summarized as follows.

1. The results of a large number of numerical calculations and analyses indicate that the support is similar to the working face and also has a constraint effect on the surrounding rock. When using the 2D tunnel model, the virtual supporting force generated by the constraint effect of the support structure should be considered, and the supporting force of the support should be increased by 2%–3% of the maximum supporting force.
2. The influence of the model boundary effect is expanded owing to the absence of a support structure at the boundary. The stress of the support structure at the model boundary is appreciably greater than that in the interior. A comparison of the field monitoring data of the Baoshan tunnel with the numerical calculation results shows that the internal force of the support structure at the model boundary is significantly greater than the actual force, and its influence range is approximately 1.5 times the tunnel span. When using the 3D numerical model, the calculation results that consider the boundary effect are more in line with engineering practice.
3. The RL and RC models are developed using the FLAC^3D^ platform, and can accurately obtain the internal stress of the support structure. Laboratory tests and field monitoring data verify that the rupturable support model can reflect the stress and failure evolution characteristics of support structures in actual projects and provide effective support for the stability analysis of support structures in tunnel engineering.

## Data Availability

The datasets used or analyzed during the current study are available from the corresponding author on reasonable request.
